# On Obscure Diseases of the Brain, and Disorders of the Mind

**Published:** 1860-11

**Authors:** 


ART. III.—*On Obscure Diseases of the Brain, and Disorders of the Mind: their Incipient Symptoms, Pathology, Diagnosis, Treatment, and Prophylaxis.* By FORBES WINSLOW, M.D., D.C.L., Oxon, etc. 8vo., pp. 576. Philadelphia: Blanchard & Lea, 1860.

CEREBRAL pathology has always been environed by so much that was obscure, that the profession has looked with interest upon all attempts to fathom its profound mysteries. From the researches of experimenters and observers, the important functions which each great organ composing the nervous system performs have been in a great measure revealed, though results yet remain to be discovered in the chain of phenomena which characterize mental and moral manifestations. We have yet to discover—it is scarcely possible that the most elaborate researches which accomplished and profound intellects can bring to the elucidation of the subject can ever discover—the intimate relations between thoughts and actions, motives and judgments, the will and its accomplishment.

We may recognize anatomically each point of surface upon the cerebral organs, and seek in vain to know its functions; yet he who aims to penetrate the obscurity which surrounds the whole subject is entitled to, and receives the acknowledgment that is due to careful and industrious efforts to advance our knowledge of the unknown. If the physiologist deserves our gratitude for teaching us the mode by which we may be instructed in the functions of these organs, is he not also entitled to our commendation who points out to us the steps which we have neglected in tracing the *pathological* changes which occur, or which are manifested by peculiar alterations of mental or moral disposition, many of them so obscure to the unpracticed eye and mind of the physician that he overlooks them as undeserving of special attention, or does not notice them at all? It is to the study of these obscurities and the illustration of the difficulties which the physician has to encounter from a neglect of apparently trivial symptoms, that the work before us is devoted.

The author, Dr. Winslow, is one who has paid unusual attention to psychical investigations, especially to those of a pathological character; and who can be so well able to judge of the healthy phenomena as he whose main study has been that of mental aberrations? Such a study has often led to a complete change of views which had been long cherished, and of which we may instance the striking case of Dr. Noble, well known as for a long time one of the clearest and most ardent of the believers in cranioscopy, whose work on the brain has indeed been quoted as the most able exponent of the so-called phrenological system, but whose views experienced a total change after he had made mental maladies an almost exclusive subject of study. With a candor that did him infinite credit, he published his recantation of long-cherished opinions. Such is the change which must inevitably follow upon more intimate acquaintance with pathological phenomena which had hitherto been surrounded with a certain amount of mystery or obscurity.

The elevated and authoritative position occupied by the author of the work before us is sufficiently indicated by the vast amount of his practice, by valuable contributions to the *Journal of Psychological Medicine*, which receives his careful editorial supervision, and by the fact that the degree of D.C.L. has been conferred upon him by the University of Oxford. Nor will the present volume detract from his well-earned reputation. Perhaps no subject could be more interesting, not only to the professional, but the general inquirer; yet there is danger that the latter may become unnecessarily alarmed when he peruses the long detail of phenomena which may really indicate the incursion of cerebral disease, and yet which may occur in those who may never experience encephalic mischief. Witness, for example, the various hallucinations of the senses, some of them of the strangest character, occasionally met with, and of which many are recorded in the present volume, which certainly indicate hyperæsthesia, and always demand attention, and yet can hardly be regarded as obscure diseases of the brain, but rather perhaps as functional aberrations; probably, however, dependent upon some modification of structure, but of a transient and inappreciable character.

In a work embracing so many interesting and instructive subjects for careful study and consideration, it is of course impossible to notice each point upon which the author has dwelt. Nor shall we attempt to follow him in the minute details of facts and cases which form a prominent and attractive feature of his work. The general design of the volume may be learned from the introductory chapter, in which he dwells forcibly upon the sad consequences of the non-recognition of the precursory or premonitory symptoms of organic affections of the brain.

"How often," says Dr. Winslow, "is the physician called upon to witness the melancholy consequences, to health of body and mind, life and reason, of a neglect of well-marked premonitory symptoms of cerebral disease! . . . . Upon investigating the history of the diseases of the encephalon, how frequently does the medical man discover that positive and unequivocal symptoms of brain affection have existed, and perhaps, during the early stage, been observed for months, and in some cases for years, without exciting any apprehension on the part of the patient, his family, or his friends!" (Page 18.)

Recognizing the obscurity in which the whole subject of cerebral affections is involved, Dr. Winslow brings to their consideration all the light which observation and experience have so well qualified him to bestow upon them. He regards diseases of the nervous system as not less likely to present characteristic diagnostic symptoms than organic affections of the viscera, and while in the heart and lungs and abdominal organs no structural lesions can occur without exhibiting, at some period or other previous to dissolution, symptoms of their existence, why should it be possible for affections of the brain to exist so long undetected? Many trivial and comparatively unimportant phenomena are overlooked, which are in fact "characteristic symptoms frequently diagnostic of disease having commenced in the brain," yet they require the skillful and practiced eye and mind of a physician who has been familiar with them to give them their proper import.

The characteristic premonitory symptoms of cerebral affections form, therefore, the subject of this work, introductory, as it is, to a more full and detailed description of special forms of disease. Of the value of a knowledge and appreciation of the precursory indications of all forms of mental disorder, the forcible language of the author may be appropriately quoted, especially as the general classification of the work, and the arrangement of subjects in the most concise and intelligible mode, are embraced within the scope of these remarks.

"Let the physician then estimate, in all its vital importance, the grave necessity for prompt treatment and decisive remedial measures, when satisfied that the enemy is at the gates, and has attacked, or is on the eve of assaulting, the citadel! Under these circumstances, hesitation, delay, or procrastination, in bringing the patient within the range of curative measures, is fraught with the direst results and with the saddest consequences. Let us not willfully close our eyes to the premonitory signs, however apparently insignificant, slight, transient, and fugitive they may appear, of actual mental disorder and brain disease; for it is in this early stage when so much may be effected by judicious medical treatment to obstruct the advance of the fatal cerebral mischief.

"Having dwelt at some length on the existence of a precursory stage in all affections of the brain, and on the importance of watching for the first threatenings of incipient cerebral disorder, I propose to investigate, in detail, the general character of the premonitory symptoms of encephalic and mental disease. It will be well, however, to premise that I cannot in this work do more than generalize on this wide and expansive subject.

"When I address myself, in the succeeding volume, to the consideration of *specific types* of brain disease, it will be my object to enter more elaborately into detail, and to point out, as far as practicable, the diagnostic premonitory signs of the various organic affections of the encephalon. Many of the symptoms to which I shall refer as valid evidences of incipient brain disorder will be found common to several lesions of this organ, each presenting an essentially different aggregate group of symptoms, as well as distinctive anatomical and pathological phenomena.

"Nevertheless, I am of the opinion that a *general* description or *resumé* of the incipient signs of morbid conditions of the brain, before considering individual forms of cerebral disease, will not be without its practical value and importance. Agreeably to this arrangement, I propose to analyze the subject in the following order :—

1. Morbid Phenomena of Intelligence.
2. Morbid States of Motion.
3. Morbid Conditions of Sensation.

This classification of the subject fully recognizes the three physiological functions of the cerebro-spinal system, viz.:

$\alpha$ Thought. $\beta$ Motion. $\gamma$ Sensation.

4. Morbid Phenomena of the Special Senses, viz.:

δ Sight. η Touch.
ε Hearing. θ Smell.
ζ Taste.

5. Morbid Phenomena of Sleep and Dreaming.
6. Morbid Phenomena of Organic or Nutritive Life, viz.:

α Digestion and Assimilation. γ Respiration.

β Circulation. δ Generation.

7. General Principles of Pathology, Treatment, and Prophylaxis."—(P. 33.)

In considering the "Morbid Phenomena of Intelligence," the general postulate is advanced that all structural lesions of the encephalon, its investing membranes and blood-vessels, are associated with some derangement, modification, or altered action of the *psychical*, *motorial*, or *sensorial* functions of the great cerebral ganglia, (πρωτον Αισθητηριον,) the *sensorium commune*. And first, in regard to changes in the psychical functions, the mind may be in a state of morbid exaltation, depression, aberration, or impairment; but, before analyzing these varied conditions, the author discusses several interesting questions, to the consideration of which nearly a hundred pages are devoted. It is difficult, in a work embracing so many important points in relation to psychical phenomena, to adopt a system of classification of facts and arguments which will comprehend all that is necessary to record, without confusion and obscurity. But in taking up the consideration of a few prominent points of interest, previous to a more profound investigation of the general divisions of his subject, we think he has given the most satisfactory and intelligible mode of analyzing the obscure subjects of incipient symptoms of mental disorders. The prominent introductory questions therein discussed are—

1. The present limited knowledge of the physiology of the nervous system, and ignorance of the phenomena of mind and life.
2. Analogy between insanity and dreaming.
3. State of the mind, when passing into a condition of alienation, as deduced from the written confessions of patients after recovery.
4. Morbid phenomena of thought, as manifested during the states of transition and convalescence from attacks of insanity.

Most of these points are such as would find interested readers among the unprofessional as well as the professional, especially when couched in the appropriate language so characteristic of the work before us. In thus touching upon subjects which have been themes for metaphysical discussion for so long a period, Dr. Winslow, as a learned and observing physician, gives to them the embellishment of an extensive medical knowledge of the cerebral conditions and changes which accompany all these moral and mental manifestations. The charm of well-described cases, which always add to the attractiveness of abstruse subjects of inquiry, is abundantly evinced in the chapter on "Confessions of patients after recovering from insanity." It will be readily appreciated, that the state of the mind during insanity cannot, in a vast number of cases, be faithfully portrayed afterwards by the unfortunate sufferer. Equally interesting must be the transition state between insanity and mental health, the period of convalescence, as described in a number of cases in Dr. Winslow's work.

"I felt," says one, "as I was recovering, the delusions gradually losing their hold upon my fancy. I then began to entertain doubts as to their reality. I felt disposed to listen patiently to the judicious advice of my physician. I was no longer irritated at being told that my perceptions were false, and began to appreciate the absurdities of other patients. One fellow-sufferer, who firmly believed that he was endowed with supernatural power and divine authority, and whom I had always considered as *sane* and improperly confined, and had invariably treated with great awe and deep reverence, I now thought must be mad!"

The eccentricities of children, the evidences of a wayward disposition, and the exhibition of uncontrollable impulses in early life, are often but symptoms of incipient unsoundness of mind, which may be overlooked. Yet even with the omissions which this oversight may produce in accurate statistical tables of insanity, the prevalence of mental aberration in early life is exhibited in no insignificant proportions. As Dr. Winslow remarks, the statistics upon this subject, which have been quoted by authorities, do not exhibit so much the prevalence of insanity in very early life, as at a period when the system is more impressible to external influences, as between the ages of ten and twenty. Many of the symptoms which manifest themselves early in life may disappear, and the child, now become the man, be free for a long series of years—perhaps forever—from a recurrence.

The conclusions of Dr. Brierre du Boismont, who has paid special attention to the incursion of mental affections in children, are quoted by the author, as follows:—

"Though, in a certain number of cases, recovery takes place, the mental alienation of children and young people is a most serious disease, partly from their antecedents, and partly on account of the imperfect development of the cerebral and other organs." (P. 144.)

After insanity has been diagnosticated, and, from being latent and unrecognized, becomes unmistakably manifested, the different shapes it assumes, its medico-legal relations, etc., form interesting subjects of study. And here, perhaps, we may at once state what signification the author applies to the word "insane," and how he distinguishes that condition from mere disorder of the mind.

"I presume it to be a generally admitted axiom that the mind may be *disordered* without being *insane*, using this phrase in its strictly legal acceptation. These conditions of morbid intellect may be considered by some as only degrees of *insanity;* but I would suggest that this term be restricted to those mental disorders accompanied with positive loss of control, clearly justifying the exercise of moral restraint, and to those morbid conditions of the intellect which sanction an appeal to the protective influence of the law. In other words, I would confine my remarks to those cases in which the mind may be said to be *pathologically* disordered, but not invariably *legally* insane." (P. 148.)

The different conditions of the mind embraced in the periods of consciousness, of exaltation, and of mental depression receive ample justice at the hands of the author in three of the most valuable chapters of the work. The period of consciousness or that incipient stage of insanity in which "the patient is fully sensible of entertaining exaggerated and unnatural impressions," and "painfully recognizes the fact that insane conceptions are struggling to master his reason, obtain an ascendency over his judgment, an abnormal influence and control over his passions, and the subjugation of his instincts," is illustrated in numerous cases of interest. Under this head are included the insidious advances of morbid thought, illusions premonitory of insanity, singular examples of hallucination, and the morbid anticipations of the invasion of insanity. How difficult to analyze the obscure pathological changes which accompany this contest between states of mental and moral health or disorder!

Who so well able to describe that painful struggle, in which so often the mental powers finally succumb, as he who has watched the inception and traced the onward progress of these morbid perversions of reason and judgment from obscure symptoms which would be wholly unappreciated by others! How sensibly must the author have felt, after the enlarged experience which has given him this power of recognizing obscure perversions of the moral and intellectual powers, the sad conclusion, that "when analyzing these sad states of morbid ideas, distressing forms of disordered emotion, and painful types of excited passion, we are obliged, alas, to confess, that there is very little in connection with them to elevate, expand, and purify the taste, or to charm, captivate, and enchain the poet's fancy!" Dr. Winslow cites the history of many patients in his personal experience who had had, for a long time prior to the manifestation of insanity, singular warnings of approaching mental disorder, and thus concludes his remarks on the subject:—

"If damage is done to the delicate cerebral structure in early life by moral or physical causes, and the material lesion, whatever be its nature, is (as is usually the case) of slow and progressive growth, we can easily understand the existence of abnormal *physical* sensations within the head, as well as of morbid *mental* impressions, (engendered by changes in the nervous tissue of the brain,)

which would, in many cases, necessarily give rise to the anticipation of insanity, or to the dread of some type of disease of the brain developing itself at an after period of life." (P. 220.)

The chapter devoted to the *Stage of Exaltation* embraces the consideration of mental conditions which are naturally of a most deceptive character, such as mental acuteness, instinctive sagacity, exhibition of wit and intelligence, while it also includes the physical agitation of incipient insanity, the premonitory signs of apoplexy, and obscure cases of general paralysis. The subject therefore naturally divides itself into two heads—*psychical exaltation* and *somatic exaltation.* Cunningly concealed insanity may baffle the diagnostic efforts of the physician, and the exhibition of acuteness on the part of the patient may lead him to infer that he is not really insane. The consideration of this subject naturally falls under the head of *Exaltation*, and the author inquires whether chloroform might not be employed judiciously to decide whether lunacy does exist.

"Feigned insanity," he says, "is often unmasked by placing the patient under the influence of chloroform. Might not the same anæsthetic agent be found serviceable in analyzing a case of cunningly concealed lunacy? There can be no doubt as to the effect of chloroform in giving, in a particular type of case, great temporary prominence to insane delusions. I have occasionally observed that when it has been found necessary to administer this anæsthetic agent by inhalation to persons mentally deranged, its immediate effect has been to develop and drag from their hiding-place hallucinations that were previously if not in a latent, but faintly and feebly manifested state." . . . . "It will be important not to confound the hallucinations and illusions occasionally induced, in persons of healthy minds, by the administration of chloroform, with those that are clearly symptomatic of a state of mental derangement."

The chapter on *Impairment of Mind* embraces the discussion of three prominent subjects: general weakness of mind, morbid phenomena of attention, and morbid phenomena of memory. We are naturally led, while examining this portion of the work, to remark upon the prevalent reference of phenomena which appertain to morbid nutrition of the brain in general to softening only. Wherever impaired intellection, accompanied by defective or depraved sensation, exists, the ready diagnosis generally is, softening; and the public and the profession are apt to be satisfied when they have a name for the morbid condition. Yet the general phenomena are usually as suggestive of induration as of softening. These remarks are applicable to the following, from the work before us:—

"Among the incipient symptoms of softening of the brain and apoplexy are occasionally observed a torpor and prostration of intellect, exhibited in an inability to undertake any kind and degree of mental work. The patient complains of a deficiency of psychical power, an exhausted state of the nervous energy, and of a want of *vis;** the brain appearing to have lost its healthy tone and stamina.

"M. Gendrin says: 'Apoplectic attacks are often preceded for some days by a difficulty in executing intellectual work, by an incapacity for unusual attention, by an extraordinary irascibility, by a morbid weakness which exaggerates impressions, and produces terrors without a cause, or by unreasonable anxiety concerning ourselves or those related to us.'

"These premonitory symptoms are not demonstrable in every case of cerebral hemorrhage, for many patients appear capable of severe brain or mind work up to the moment immediately preceding the apoplectic or paralytic fit; but in many cases this conscious diminution of vigor of brain and impairment of mind are important premonitory signs of approaching acute paralytic and apoplectic seizures. The symptom, however, is present in other states of disease of the brain. Should this condition of mind be associated with giddiness, headache, depressed spirits, aberration, or impairment of vision, or a slight sensation of numbness (even if circumscribed) in any part of the body, the patient may well be anxious as to the state of his cerebral health." (P. 267.)

The *Morbid Phenomena of Memory*, which are indicative of acute diseases of the brain and disorders of the mind, are fully discussed in five chapters, embracing the general heads of Acute and Chronic Disorders of Memory, Perversion of Memory, Exaltation of Memory, Memory of the Insane, and Psychology and Pathology of Memory. It would be impossible to follow the author in his minute divisions of the subject, which is abundantly illustrated by cases in his own experience and that of others. In his chapter on the Psychology and Pathology of Memory, Dr. Winslow considers the difficult subject of the mode in which sensational and intellectual phenomena are effected. That the impression is recorded in the brain, and in the varying nutrition of the organ the impression is repeated on the new matter, can scarcely be contested. Of such repetition in disease we have examples in the cachexiæ, in the cancer cell, for instance, which continues to produce its like; but the morbid condition is in the structure, not in the blood. We cannot, therefore, agree with Dr. Winslow in the pathological inference which he has emphasized by italics in the following quotation. The impression in such case is, we think, undoubtedly in the structure—in the cells of nutrition—the blood only acting as a pabulum.

"If we turn to the consideration of pathological phenomena, the physiologist is still more bewildered in his attempt to penetrate behind the veil that conceals from finite understandings the incomprehensible laws regulating the operations of life, as dependent upon and connected with the organization of the body. I refer to those subtle changes in the character of the blood effected in infancy by

* We hear this in common parlance most erroneously called *vim*, and often by those who ought to know better; as, "He is a person of great *vim!*"

the introduction into it of minute portions of morbific matter with a view of protecting the body from the influence of noxious and often deadly poisons. I allude to the effect of the vaccine virus upon the blood in producing a *permanent and organic change in its constitution and character, which continues to exercise a protective influence against small-pox in the great mass of cases, through a long life, during which time the blood must have undergone many thousands, if not millions, of changes and modifications!* If we could imagine a person so armed, by means of the introduction into the system of healthy vaccine matter, under favorable bodily conditions for its reception, to be drained of nearly his last drop of blood, and subsequently restored to his original vascular condition, we should find no diminution in the force of its sanitary effect upon the vital fluid in early life; in other words, he would continue protected certainly for many years, from the influence of the small-pox poison." (P. 349.)

We wish we could agree with Dr. Winslow in the hope expressed in the following paragraph. The microscope cannot carry us much farther, perhaps, than it has already done; while organic chemistry can only enlighten us on the dead, certainly not on the living cerebral substance.

"In the present imperfect state of our knowledge of the intimate character and composition of nerve structure, admitted ignorance of the *vis nervosa*, as well as of the laws governing the operations of thought, as connected with and dependent upon recondite alterations in the vesicular neurine of the brain, it would be useless to speculate as to the cause of the psychical phenomena to which I am about to refer. Much light may yet be thrown upon this important and intricate subject, as the result of a closer study of mental dynamics as well as of *chemico-cerebral* pathology. Morbid phenomena of mind, incomprehensible to the physiologist, and inscrutable to the pathologist, may be intimately dependent upon minute changes (out of the range of the microscope, and only to be detected in the laboratory) in the organic chemical constitution of brain-matter affecting not only the *quantity, quality*, but distribution of the *nerve* and *psychical* force, not on the existing state of our knowledge of physiological and dynamical science, susceptible of demonstration." (P. 351.)

The indestructible character of ideas, which forms an essential feature of the process of memory, is exhibited under special conditions in a marked manner. For example, in certain forms of asphyxia, caused by drowning and hanging; in conditions of the mind preceding death; and in morbid mental phenomena observed to result from injuries to the brain, or to be consequent on special forms of encephalic disease. The sensations of drowning, as cited in cases furnished by the author, afford an exemplification of the hold which ideas once fixed have upon the mind.

"They were the most delightful and ecstatic I have ever experienced. I was transported to a perfect paradise, and witnessed scenes that my imagination never had, in its most active condition, depicted to my mind. . . . . While in this world of fancy, my mind had recalled to consciousness the scenes and associations of my early life, and the memory of the companions of my boyhood. All the knowledge I had acquired during a long life recurred to my mind. Favorite passages from Horace, Virgil, and Cicero were revived, and pieces of poetry I had been fond of repeating when a boy, came fresh to my recollection."

And this case naturally leads to the examination of the singular fact that severe illness often revives the knowledge of languages which had been apparently totally forgotten for years. The highly interesting case detailed by Coleridge, in which a young woman, who could neither read nor write, but who, when attacked by fever, talked incessantly Latin, Greek, and Hebrew, is a remarkable illustration of the tenacity with which impressions once made often remain fixed in the mind. Years before she had been employed as a domestic in the family of a clergyman who was a great Hebraic and classical scholar.

Dr. Winslow, in referring to the able researches of Dr. Draper* upon impressions made upon *material* substances cognizable to sense, inquires whether such experiments can "throw any light upon the *physical* or *psychical* phenomena of memory."

"If," says Dr. Draper, "on a cold polished piece of metal any object, as a wafer, is laid, and the metal then be breathed upon, and, when the moisture has had time to disappear, the wafer be thrown off, though now upon the polished surface the most critical inspection can discover no trace of any form, if we breathe upon it, a spectral figure of the wafer comes into view, and this may be repeated again and again. Nay, even more; if the polished metal be carefully put aside where nothing can deteriorate its surface, and be so kept for many months, even for a year, on breathing again upon it, the shadowy form emerges; or, if a sheet of paper on which a key or other object is laid, be carried for a few moments into the sunshine and then instantaneously viewed in the dark, the key being simultaneously removed, a fading spectre of the key upon the paper could be seen, and if the paper be put away where nothing can disturb it, and so kept for many months, at the end thereof, if it be carried into a dark place and laid upon a piece of hot metal, the spectre of the key will come forth. . . . . A shadow is said never to fall upon a wall without leaving thereupon its permanent trace, a trace which might be made visible by resorting to proper processes. All kinds of photographic drawings are, in their degree, examples of this kind. Of the moral consequences of these phenomena, it is not my object here to speak."

How analogous the action of memory to the physical actions so well described by Dr. Draper!

The chapter on *Morbid Phenomena of Motion* is especially valuable to the physician, as pointing out the premonitory symptoms of apoplexy and paralysis, irregularities of muscular action, and insidious symptoms of epilepsy, which, once recognized, become important elements of consideration

---

* Human Physiology, Statical and Dynamical, p. 288.

in a diagnostic point of view. Under the division of *convulsive action*, embracing "those irregular and morbid states of the motor power or muscular fibre generally grouped under the head Convulsions," epilepsy receives special attention. Discarding the classification of epileptic diseases, which is usually adopted by writers, Dr. Winslow finds much greater facility in describing the different forms of the disease in the following threefold division.

*a. Epilepsy.*

(With violent muscular movements.)

*β. Epilepsy.*

(Nocturnal in its character, and accompanied with slight muscular convulsions.)

*γ. Epileptic Vertigo.*

(Without muscular convulsions.)

It will be seen by this arrangement that Dr. Winslow adds to the *Petit Mal* and *Grand Mal* of the French, a modified form of epileptic attack which usually occurs at night.

In connection with the subject of *Morbid Phenomena of Speech*, we may refer to the important premonitory symptoms of cerebral disease, characterized by irregular action of articulation, in which, in the words of the author, there is a want of co-ordination in the action of those portions of the nervous centres necessary for the production of articulate sounds, or, more correctly speaking, as suggested by Romberg, "there exists an interruption (caused by various morbid states of the brain) in the pre-established harmony which should obtain between the subjective intelligence and the organs of speech, giving rise to those singular anomalies in the co-ordinating faculty of articulation occasionally witnessed in connection with organic cerebral conditions." The curious phenomena presented by alterations of speech are fully discussed and illustrated by the author. They are frequently the most difficult to explain; for it is almost impossible to appreciate why the memory of certain words only should be lost, while the reasoning faculties seem to be intact; and why certain words should be substituted for others.

But we have not space for further comments. We merely refer to the subjects embraced in the remaining chapters as exhibiting the general character of the work. They are fully as important to medical men as guides to the recognition of obscure disorders of the mind as those which have preceded, considering as they do the morbid phenomena of sensation, of vision, hearing, taste, touch and smell; of sleep and dreaming, of organic and nutritive life; and finally, a very full view of the general principles of pathology, diagnosis, treatment, and prophylaxis of cerebral diseases—all of which form, with the portions of the work previously considered, a general introduction to a treatise on "Organic Affections of the Brain," and to another on "Disorders of the Intelligence, Cerebro-Psychical in their Nature." The volume concludes with a beautiful picture of the qualities which a psychological physician should possess to constitute him worthy of his position.

The work should be in the possession of every physician. It is full of details of the deepest moment; and, while strictly historical, has all the charms of the most interesting novel, so that when its perusal has been once commenced, it is with reluctance abandoned. From the first portion of the Introduction, the reader might be disheartened by the hackneyed exordium in Greek characters to the Aphorisms of Hippocrates. It is the only parade of learning, however, in the work. The style is as attractive as it is accurate. We were at first, indeed, slightly puzzled by the reference to Professor Kolk, and doubt whether Schroeder Van der Kolk ever heard himself so designated. We have seen Parent du Chatelet designated as Duchatelet. His proper name, however, is Parent, and du Chatelet is the distinctive, as is Van der Kolk. We only recollect at this time one case in which the distinction has been sanctioned, that of Du Fresne, Dominus du Cange—now better, indeed almost universally, known as Du Cange.

